# Does recognized genetic management in supportive breeding prevent genetic changes in life-history traits?

**DOI:** 10.1111/eva.12150

**Published:** 2014-03-27

**Authors:** Rémi Chargé, Gabriele Sorci, Michel Saint Jalme, Loïc Lesobre, Yves Hingrat, Frédéric Lacroix, Céline Teplitsky

**Affiliations:** 1 Centre d'Ecologie et de Sciences de la Conservation UMR 7204 CNRS/MNHN/UPMC, Muséum National d'Histoire NaturelleParis, France; 2 Emirates Center for Wildlife Propagation (ECWP), Province de BoulemaneMissour, Morocco; 3 Centre of Excellence in Biological Interactions, Department of Biological and Environmental Science, University of JyväskyläJyväskylä, Finland; 4 Biogéosciences, UMR CNRS 6282, Université de BourgogneDijon, France; 5 Reneco Wildlife Consultants LLCAbu Dhabi, United Arab Emirates

**Keywords:** captive populations, conservation genetics, quantitative genetics

## Abstract

Supportive breeding is one of the last resort conservation strategies to avoid species extinction. Management of captive populations is challenging because several harmful genetic processes need to be avoided. Several recommendations have been proposed to limit these deleterious effects, but empirical assessments of these strategies remain scarce. We investigated the outcome of a genetic management in a supportive breeding for the Houbara Bustard. At the phenotypic level, we found an increase over generations in the mean values of gamete production, body mass and courtship display rate. Using an animal model, we found that phenotypic changes reflected genetic changes as evidenced by an increase in breeding values for all traits. These changes resulted from selection acting on gamete production and to a lesser extent on courtship display. Selection decreased over years for female gametes, emphasizing the effort of managers to increase the contribution of poor breeders to offspring recruited in the captive breeding. Our results shed light on very fast genetic changes in an exemplary captive programme that follows worldwide used recommendations and emphasizes the need of more empirical evidence of the effects of genetic guidelines on the prevention of genetic changes in supportive breeding.

## Introduction

Because of human activities leading to habitat loss, overexploitation, climate changes and spread of invasive species, we are currently facing a so-called sixth extinction (Barnosky et al. 2011), with current species loss being 100 to 1000 times faster than previous mass extinctions (Pimm et al. 1995). The urgent need for efficient conservation strategies has resulted in an increasing number of areas where biodiversity is preserved. However, threats are sometimes difficult or even impossible to remove (e.g. habitat loss and climate change), leading to implement *ex-situ* conservation policies to mitigate species loss. Among these *ex-situ* programmes, supportive breeding is used when the species habitat is still available, but wild populations cannot sustain themselves (e.g. because of overexploitation). The goal of supportive breeding programmes is therefore to increase the effective size of wild populations through release of captive-born individuals (Wang and Ryman 2001; Duschesne and Bernatchez 2002; Wedekind 2002; Blanchet et al. 2008), which should substantially decrease extinction risk. As such, captive breeding is a widely used tool to restore populations of threatened species (Allendorf and Luikart 2007; Frankham 2008).

Genetic breeding programmes rest on specific guidelines to avoid genetic changes in captive populations (Frankham et al. 2000; Wang and Ryman 2001; Fraser 2008; Williams and Hoffman 2009). For example, genetic drift, stronger in small populations, may lead either to the loss or to an increased expression of rare alleles, which in the latter case could be dramatic when these alleles have deleterious effects. Hence, management strategies need to take into account the emergence of a certain genetic load in captive populations (Grahn et al. 1998; Lacy 2000; Lynch and O'Hely 2001; Wedekind 2002; Pitcher and Neff 2007) that could be transferred to the reinforced wild populations (Reisenbichler and Rubin 1999; Woodworth et al. 2002; Araki et al. 2009).

One strategy to limit these genetic changes is to equalize the representation of each founder in the captive population (Ballou and Lacy 1995; Frankham et al. 2000; Lacy 2000). To this end, managers mate pairs based on their relatedness (mean kinship) assessed from pedigree analysis. Mean kinship is high when individuals are over-represented in the population and low when individuals represent rare founder genetic lines (Ballou and Lacy 1995; Grahn et al. 1998; Saura et al. 2008; Asa et al. 2011). Together with the integration of founders individuals (Frankham and Loebel 1992), these breeding methods are assumed to halve the rate of genetic adaptation to captivity (Frankham and Loebel 1992; Saura et al. 2008), reduce drift and maintain genetic variation.

However, equalizing founder lines in captive breeding may be jeopardized if individuals from rare founder lines contribute little to reproduction. Variation in breeding success directly creates opportunities for unintentional selection (Williams and Hoffman 2009). To circumvent unbalanced contribution in the offspring, one technique consists in the genetic dumping strategy in which offspring from the most represented captive breeders (i.e. with the highest mean kinship) are preferentially released in the wild, when a reinforcement programme is associated with the supportive breeding (Earnhardt 1999).

By 2003, 489 reintroduction projects in animal species were implemented (Seddon et al. 2005), but empirical studies of the impact of breeding programmes on genetic changes are still rare (but see the review from Williams and Hoffman 2009), although powerful tools such as quantitative genetics can provide some clear answers (Pelletier et al. 2009). More specifically, the *animal model* is a statistical method that allows the estimation of individual breeding values (i.e. genetic value of an individual for a given trait), so that testing for trends in these genetic values will inform us on potential genetic changes. New methods even allow assessing the strength of these trends compared with expectations based on drift only (Hadfield et al. 2010).

In this paper, we investigated the efficiency of a breeding programme that has used genetic dumping strategy and regular integration of new founders to the captive flock of breeders by analysing data from a captive population of Houbara Bustard (*Chlamydotis undulata*) (Lesobre 2008). We estimated genetic changes in heritable, fitness-related, traits potentially occurring in a supportive breeding aiming at safeguarding that bird species.

## Material and methods

### Breeding programme

The North African Houbara Bustard is a middle-size bird. Males are sexually mature when they are 2–4 years old and females when they are 1–2 years old. The mating system of the Houbara is a dispersed lek, with males performing a courtship behaviour at display sites during the breeding season (from January to June) to attract females. Males provide females with nothing, but genes through the semen. Females generally lay clutches from one to four eggs.

The Houbara Bustard is endangered across all its distribution area mainly due to overhunting and habitat degradation (Goriup 1997), leading to the creation, in 1996, of a captive breeding in Morocco to supplement North African wild populations (Lacroix 2003). The first eggs were collected in 1986 and 1987 in Algeria and transferred to the National Wildlife Research Center (Taïf, Saoudi Arabia, Saint Jalme et al. 1996). These founders and their offspring (*n* = 296) were transferred in Morocco to the Emirates Center for Wildlife Conservation in 1996. Two campaigns for egg collection were conducted in Morocco in 1996–1997 and between 2002 and 2008. In total, 564 chicks from wild-laid eggs were integrated to the captive breeding by 2009 (Table 1).

**Table 1:** (a) Number of chicks from wild-laid eggs added to the captive population each year. (b) Yearly production of captive chicks either integrated to the captive population or released in the wild

Year	Number of chicks
(a)	
1986	31
1987	39
1996	27
1997	57
1998	2
2001	2
2002	71
2003	96
2004	106
2005	24
2006	1
2007	75
2008	31
2009	2
Total	564

Year	Integrated	Released
(b)		
1997	98	67
1998	69	58
1999	79	69
2000	302	127
2001	253	157
2002	493	300
2003	475	385
2004	1047	1104
2005	504	1544
2006	642	3223
2007	1065	7081
2008	1062	7168
2009	1832	14 790
2010	610	14 385
2011	766	13 968
Total	9297	64 426

Breeding birds were housed outdoor in individual cages (2 × 4 m²). Food and water were provided daily *ad libitum*. Females were artificially inseminated with semen from males depending on their mean kinship (Saint Jalme et al. 1994). Males were collected for semen every 2 days on average, using a dummy female. Semen was immediately transferred into a vial and diluted in Lake 7.1 diluent (Lake and Ravie 1984; Saint Jalme et al. 2003). Semen was subsequently used to inseminate females according to the genetic management programme aiming at equalizing the founders’ contribution and avoiding inbreeding. Eggs are collected to stimulate several replacement clutches, leading to an average production of six eggs per female per year. Eggs laid were collected every day and transferred to an incubator in standard conditions over the incubation period of 23 days. At hatching, chicks were transferred to a rearing facility and hand-fed. To implement genetic management of the captive population, chicks sired by the most represented breeders in the captive flock (i.e. with the higher mean kinship) were preferentially released in the wild for the reinforcement of wild populations (i.e. the genetic dumping strategy Lesobre 2008). Furthermore, the regular addition of founders was used to increase the genetic diversity of the captive flock. Generations were all crossed leading to a mismatch between generations and cohorts (Lesobre 2008).

### Measured traits

Courtship display, ejaculate size, number of eggs laid and body mass per year were analysed to assess change in breeding values over time. Measurements of these traits were available for thousands of birds with a known pedigree that reached 74 528 individuals in 2011. Full statistics on pruned pedigrees used in analyses are given in Appendix S1. We used phenotypic data collected from 1999 to 2011 on 3230 males and 5201 females born in 1986 and onwards.

Body mass (±1 g) was measured in both males and females several times per year. Because body mass shows some within-year variation (Saint Jalme et al. 1996), we yearly corrected each measure by the day of measurement (R software, *lmer* function: body mass ∼ day + day^2^ + 1|bird identity + 1|year, with day 1 = 1 January).

Houbara sexual display is characterized by a complex behaviour including a circular running with the white feathers on the neck and the head fully erected. During the breeding season, males devote several hours per day to courtship activity (Hingrat et al. 2008). Sexual display was recorded by staff members of the ECWP during three daily scans (at dawn, morning and afternoon before 2010 and only at dawn afterwards). ECWP staff moved around individual cages and scored the presence or absence of courtship display for each male. A score of 1 was assigned to a male when it was displaying during at least one behavioural scan; otherwise a score of 0 was set. The first scan of the day was considered to capture most of the daily interindividual variation because 98% of males that were displaying during the morning were also displaying at dawn. Total numbers of days with display were summed by year. A missing value was assigned for years preceding the first observation of display in male life. Otherwise, a zero was set for years where male has not been seen displaying during the whole year.

Ejaculate size was assessed as the number of spermatozoa per ejaculate using a spectrophotometer at a wavelength of 600 nm. Mean number of spermatozoa was then calculated yearly.

We used number of eggs laid per breeding season as a proxy of female fecundity. Likewise courtship display, a zero was assigned for years without any egg, except for years preceding the first breeding season in a female lifetime.

### Calculation of selection coefficients

We estimated selection coefficients using a linear regression between traits and relative fitness (i.e. individual fitness divided by population average) as described by Lande and Arnold (1983). Because a dumping strategy is in use in this breeding programme, fitness was estimated by the number of chicks recruited to the captive breeder flock and not the total number of offspring produced. To compare patterns of selection between traits, phenotypic values were standardized (mean = 0, SD = 1) within years.

We first estimated selection differentials for each year using linear regressions with a normal distribution of errors (R software, *lm* function) because estimates are not affected by the non-normality of data (Lande and Arnold 1983). However, to test the statistical significance of models, a second regression model was performed on nonstandardized values of number of chicks with a Poisson distribution of errors (R software, *glm* function). Age and a squared age were set as fixed factors in the models.

Selection can directly target one trait and indirectly produce a selection pattern on a correlated trait. Because traits investigated here have been shown to be correlated both at the phenotypic and genetic levels (Chargé et al. 2013), we also ran two additional models to estimate selection gradients in males and females that were including for males display rate, ejaculate size and male body mass, and for females number of eggs and female body mass.

To assess a global selection gradient, we used the meta-analysis implemented in MCMCglmm where an estimate-specific measurement error can be included. For each trait, the model was


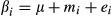
(1)

Where *β* is the selection gradient, *μ* is the intercept, and m the error associated with *β* and e the residuals. The random effects are assumed to follow normal distributions with 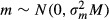 where 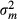 is the measurement error, and *M* is a diagonal matrix where each element is the square of the standard error. The variance 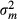 is fixed to 1. The errors follow the distribution 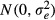. Because of a convergence issue for selection gradients on courtship display rate and male body mass, we included a prior for intercept with *μ* = 0 and *V* = 200, where *V* is the variance of the prior. *V* is large so that the prior is diffuse and weakly informative.

Selection intensity is more likely to vary among years (e.g. due to changes in breeding practices) than according to generations. Therefore, to assess for temporal trends in selection, we used the meta-analysis implemented in *MCMCglmm* similar to (1) but including Year as a linear fixed effect:


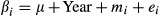
(2)

### Phenotypic changes

Phenotypic trends were assessed with a mixed model for each separate trait. Because trait values change with age, we included age and age² effects. The trend was assessed across generation, not years, so that generation was included as a continuous variable and year as a random effect. To account for repeated measurements, we also included individual identity as a random effect. A normal distribution was assumed for body mass and a Poisson distribution for number of eggs, display rate and ejaculate size (*MCMCglmm* package).

### Quantitative genetic analyses

To estimate breeding values, and thus the trends at genetic level, we fitted an individual animal model (Lynch 1998; Kruuk 2004) for courtship display, ejaculate size, body mass and female fecundity. The model uses information from pedigree and phenotypic values to decompose the phenotypic variance of a trait into its additive genetic variance and other components of variance. Age of birds and its quadratic term were included as fixed factors to take into account any effect of immaturity and/or senescence on reproductive traits (Preston et al. 2011). Removing age from fixed effects did not affect consistently the estimation of the additive genetic variance in our models (Supporting information, Table S5). It has been shown that frequency of sperm collection does not influence additive genetic variance of sperm count (Chargé et al. 2013). Therefore, this factor was not taken into account in our models. Year was fitted as a random factor into the model to control for interannual environmental variation. Individual identity was fitted as a factor linked to the pedigree to estimate additive genetic variance and breeding values. Permanent environment (identity effect not linked to the pedigree) was included to account for repeated measurements on the same individual (Kruuk 2004). Maternal effects were not included in the following model because there was no significant effect on the estimation of genetic additive variance (Supporting information, Table S5).

In matrix notation, for each trait the model is specified as follows:


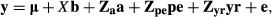
(3)

where **y** is the vector of phenotypic observations for all individuals, ***μ*** is the mean phenotype, and **b** is the vector of fixed effects to be fitted (age) and ***X*** the design matrix relating phenotypic observations to the vector of fixed effects. For the random effects, **a** is the vector of additive genetic values, **pe** the vector of permanent environment effects, and **yr** the vector of year of measurement effect, with **Z**_a_, **Z**_pe_ and **Z**_yr_ their respective incidence matrices. All random effects are assumed to be normally distributed, and elements of **a** are assumed to be drawn from 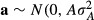), where 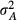 is the additive genetic variance, and A the relatedness matrix derived from the pedigree.

The animal models were run using the Bayesian method (R software, MCMC package, Hadfield et al. 2010). The advantages of the Bayesian method are twofold: (i) it estimates the whole posterior distribution of estimated effects, including breeding values; (ii) it allows fitting non-normal distributions as required for courtship display, number of sperm and eggs. For these Poisson traits, breeding values were back-transformed using an exponential function.

In contrast to traditional REML methods, using regression of breeding values based on posterior distribution allows a conservative estimate of evolutionary trend (Hadfield et al. 2010). Moreover, a method implemented in MCMCglmm allows assessing the strength of the trend compared with what could be expected under genetic drift only. More specifically, for each iteration from the model, we computed the average breeding values per generation of (i) actual estimated breeding values from the population and (ii) simulated breeding values under drift (*rbv* function in MCMCglmm). The slope of the regression between average breeding values against generation is stored at each iteration for both actual (reg1) and simulated (reg2) breeding values. The slope of the genetic trend in the captive population is the posterior mode of reg1. The significance of the trend is based on the number of times the trend is superior or inferior to 0, depending on the sign of the posterior mode. The comparison of the genetic trend to expected trend under drift is simply the number of times the trend from reg1 is superior to the trend from reg2.

Note that here, in contrast with studies in wild populations, trends are calculated over generations and not according to year of birth. The posterior distribution of breeding values was a sample of 1000 values for each parameter: we used a total of 1 200 000 iterations for each analysis, with a burn-in phase of 200,000 and thinning of 1000. We assessed two priors for variances (*V*_A_, *V*_PE_ and *V*_YEAR_) for each analysis: (i) a parameter-expanded prior (Gelman 2006), which is weakly informative prior of the shape (*V* = 1, *η* = 1, *α*.*μ* = 0 and α.*V* = 100 000), and (ii) a slightly informative prior (*V* = *V*p/r, *η* = 1), where *V*p is the phenotypic variance and *r* the number of random factors. Note that the prior for *V*_R_ is (*V* = *V*p/*r*, *η* = 1) in both cases. Our results were not sensitive to the choice of priors (Supporting information, Table S4), and results presented in Table 2 were obtained under the parameter-expanded priors. We also presented trends in breeding values standardized in Haldanes (in units of standard deviation, Table 2).

**Table 2:** (a) Variance components in animal models with 95% confidence interval for additive genetic variance (*V*_a_), permanent environment variance (*V*_pe_), year variance (*V*_year_) and residual variance (*V*_r_). Normal scale has been used for body mass and Poisson latent scale for courtship display, ejaculate size and number of eggs. (b) Time trends in breeding values compared with trend expected under drift. Table presents the estimates with 95% confidence interval and the probability of the posterior distribution for the estimate being equal to zero (*P*_T_), and similar to the expectation under the hypothesis of drift only (*P*_D_). Haldanes represent a standardized change in breeding values

	*V*_a_ [95%CI]	*V*_pe_ [95%CI]	*V*_year_ [95%CI]	*V*_r_ [95%CI]
(a)				
Courtship display	1.2 [1.2; 1.3]	1.3 [1.3; 1.4]	1.1 [1.0; 1.3]	2.0 [1.9; 2.0]
Ejaculate size	1.3 [1.2; 1.5]	1.3 [1.2; 1.4]	1.0 [1.0; 1.2]	1.3 [1.2; 1.3]
Number of eggs	1.2 [1.2; 1.3]	1.2 [1.1; 1.2]	1.1 [1.0; 1.2]	1.2 [1.2; 1.2]
Female body mass	10 726.3 [9574.8; 12 258.0]	5083.7 [4487.9; 6036.2]	1336.6 [575.0; 3879.3]	3830.8 [3749.2; 3921.9]
Male body mass	21 122.7 [18 118.8; 24 723.5]	11 998.0 [10 315.6; 14 374.9]	902.8 [354.9; 5704.9]	9567.8 [9224.3; 9812.8]

	Trend in breeding values estimate [95% CI]	*P*_T_	*P*_D_	Haldanes (SD per generation)
(b)				
Courtship display	0.27 [0.21; 0.42]	0	0	0.006
Ejaculate size	0.17 [0.06; 0.31]	0	0.024	0.010
Number of eggs	0.21 [0.18; 0.30]	0	0	0.037
Female body mass	19.04 [14.67; 21.86]	0	0.001	0.128
Male body mass	37.35 [29.34; 44.89]	0	0.001	0.173

## Results

### Selection

Global selection differentials were always positive ranging from 0.04 for male body mass to 0.72 for number of eggs and always significantly different from zero except for male body mass (Table 3). Interestingly, global selection gradient for female body mass was not significantly different from zero contrary to selection differential, while selection differentials and gradients for number of eggs were similar (Table 3), suggesting direct selection acting on female fecundity, but indirect selection on female body mass. Selection acting on male traits was the strongest for ejaculate size and the weakest for body mass (with intermediate values for display rate).

**Table 3:** Global selection differentials and gradients in the breeding facility, estimated from annual selection estimates

	Global selection estimate [CI]	Probability to be equal to 0
Global selection differentials		
Courtship display	0.19 [0.13;0.26]	**0.01**
Ejaculate size	0.51 [0.37;0.66]	**<0.005**
Male body mass	0.04 [−0.03;0.11]	0.29
Number of eggs	0.72 [0.57;0.89]	**<0.005**
Female body mass	0.23 [0.10;0.36]	**0.01**
Global selection gradients		
Courtship display*	0.16 [0.07;0.22]	**<0.005**
Ejaculate size*	0.45 [0.34;0.58]	**<0.005**
Male body* mass	−0.03 [−0.10;0.05]	0.52
Number of eggs†	0.71 [0.56;0.81]	**<0.005**
Female body mass†	0.04 [−0.04;0.11]	0.33

* Selection gradients from models in which courtship display, ejaculate size and male body mass have been set as covariates.

† Selection gradients from models in which number of eggs and female body mass have been set as covariates.

Bold values refer to p < 0.05.

**Figure 1:**
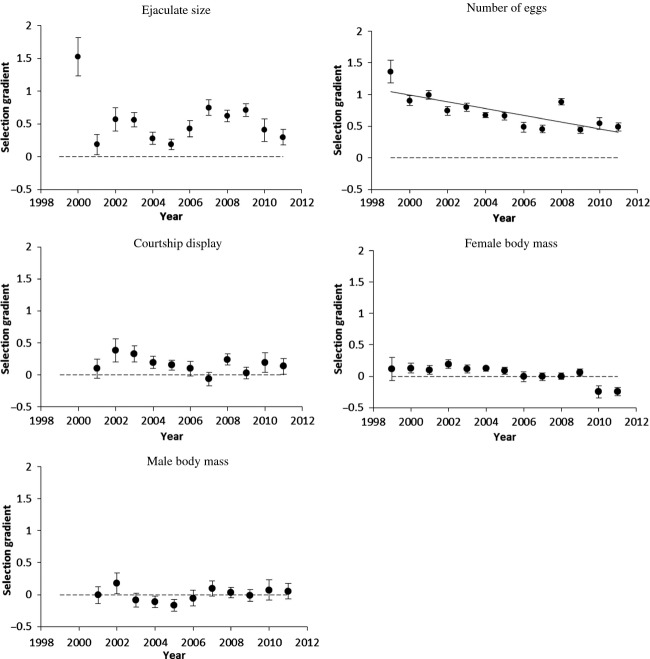
Trends in standardized selection gradients. Bars represent standard errors. Solid lines show significant trends of selection over years, and dashed lines represent zero selection.

The strength of selection was variable according to the year of breeding but overall, selection decreased with time in females (trends in number of eggs: −0.05 [−0.07; −0.02], *P* < 0.005), but not in males (Fig. 1, Supporting information, Tables S2 and S3). However, selection gradient on courtship display rate was very low except for 2002 and 2003. Given that all these traits are known to be heritable (Chargé et al. 2013), a genetic response to selection is expected and should result in changes in the breeding values.

### Breeding values

**Figure 2:**
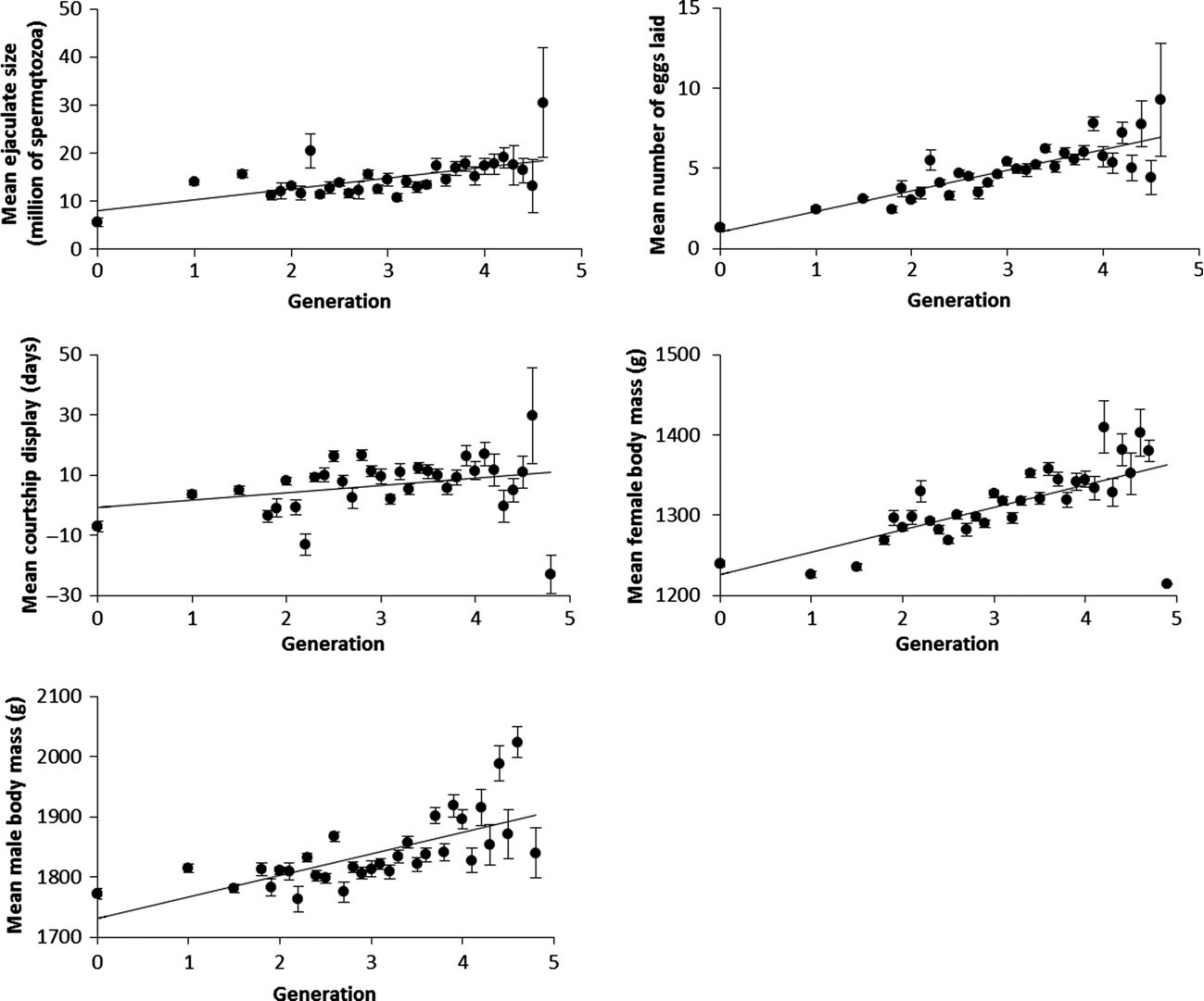
Trends in phenotypic changes. Bars represent standard errors. Solid lines show significant phenotypic changes. Values are corrected by age and quadratic age of individuals.

Phenotypic values were significantly increasing over generations for all the traits assessed (Table 4, Fig. 2).

**Table 4:** Time trends in phenotypic values over generations. Table presents the estimates with 95% confidence interval. Normal scale has been used for body mass and Poisson latent scale for courtship display, ejaculate size and number of eggs

	Trend in phenotypic values [95%CI]	Probability to be different from 0
Courtship display	0.23 [0.20; 0.26]	<0.001
Ejaculate size	0.18 [0.14; 0.22]	<0.001
Number of eggs	0.19 [0.17; 0.21]	<0.001
Female body mass	19.6 [15.44; 23.548]	<0.001
Male body mass	33.80 [26.71; 40.80]	<0.001

**Figure 3:**
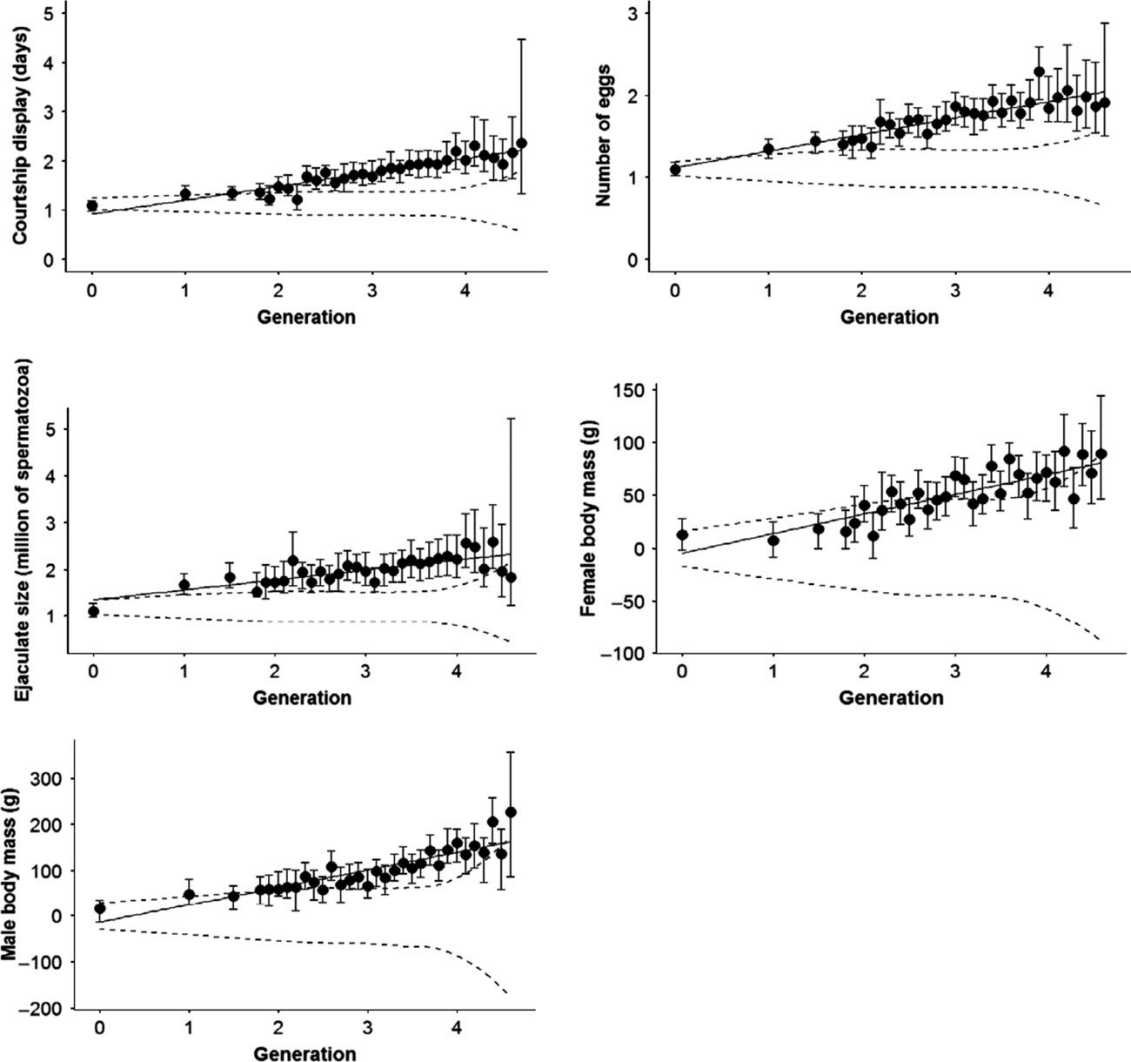
Trends in breeding values. Filled points represent posterior modes of breeding values averaged per generation, with confident intervals. Solid regression line represents the trends in the breeding values and the trend expected under drift only. Dashed lines refer to changes under genetic drift (confident intervals).

There was a significant increase in breeding values over generations for all traits, including body mass in spite of no direct selection on this trait. Trends in breeding values were always larger than what could be expected based on drift alone (Table 2, Fig. 3). In four generations of captivity average breeding values increased of 1.1 days for number of days with display, 0.68 million spermatozoa for ejaculate size and 0.84 eggs for number of eggs laid. The standardized Haldanes showed quite moderate response to selection (Table 2). Surprisingly, trends were strongest for body mass, in spite of the absence of direct selection, probably because of their higher heritability.

## Discussion

We aimed to assess the efficiency of worldwide management recommendations of captive populations for conservation purposes. This is, to our knowledge, one of the first studies investigating genetic changes in reproductive, behavioural and morphological traits in a large captive population of vertebrates, under a strict genetic management that follows well-established guidelines (Frankham et al. 2000). Despite the concerns about genetic changes in captive breeding, most of, if not all, empirical studies have assessed genetic changes in captive populations not submitted to carefully planned genetic management (but see for instance Frankham et al. 2000 for a specific assessment of equalization of family size and Earnhardt 1999 about the genetic dumping strategy). We believe that our results shed light on the evolutionary processes occurring in supportive breeding and have important implications for improving such conservation tools.

The Houbara supportive breeding managed by ECWP was a unique opportunity to investigate potential genetic changes because the captive population was established ∼70 founders, regularly reinforced by wild-laid eggs (∼500), leading to a very large captive population of about 10 000 adult breeders (∼7000 still alive in 2012), reared and bred individually for 15 years (∼5 generations) so that impressive pedigree records and precise phenotypic dataset were available. The aim of the Houbara breeding management was fourfold: (i) avoiding inbreeding, (ii) equalizing the representation of each founder line by forming pairs based on their mean kinship, (iii) maintaining high genetic diversity by regularly integrating new founders, from wild-laid eggs, to the captive broodstock and (iv) limiting best breeders to be over-represented in the captive population by preferentially releasing in the wild offspring sired by the most represented breeders (i.e. the genetic dumping strategy).

We show here, using recently implemented quantitative genetics tools, evidence that genetic changes can occur despite these strict guidelines, although consequences do not lead inevitably to lower fitness in the Houbara Bustard.

### Selection patterns

We found that global selection gradients were higher for ejaculate size and number of eggs (0.45 and 0.70, respectively) than for courtship display (0.16) and body mass (−0.02 in males and 0.04 in females). Statistically significant selection coefficients of reproductive traits indicate that the best captive breeders did contribute more to the number of recruited offspring, despite the effort produced to equalize the representation of each founder in the captive population.

The difficulty to limit contribution of prolific breeders might stem from the so-called growth phase that represents the early stage of captive breeding during which managers have to increase captive population size to rapidly set up a sizeable flock of adult breeders. During this period, demographic goals might have prevailed over genetic ones. In line with this, there is a strong disequilibrium in founder representation, with only 19% of founders representing up to 83.5% of the genetic variability of the captive population in 2007 (Lesobre 2008).

The difficulty to limit contribution of prolific breeders to the next generation is probably very common in supportive breeding for species with strong reproductive skew. The situation seems difficult to solve because poor breeders will never achieve a great contribution to the next generation. Surprisingly, despite similar issues may be very common in many captive breeding, empirical evidence of such phenomenon is very hard to find in the literature.

One solution to improve the contribution of poor breeders from rare genetic lines could be to reduce the growth speed of captive populations to enable managers to better control the contribution of different individuals to the next generation. However, the implementation of such strategy is challenging as threatened species usually suffer from a fast decline in the wild, which may preclude any attempt to decrease the speed of the population's growth in captivity. Breeding rapidly numerous individuals is required to prevent wild-supplemented populations from losing genetic diversity or even from extinction (Ralls et al. 2000).

### Genetic changes

Consistent with the strength observed in selection gradients and the moderate heritability of these traits (Chargé et al. 2013), we found significant genetic changes for all the investigated traits (courtship display in males, gametes number and body mass) over the four generations covered by the study (13 years of data, 23 cohorts).

Trends in breeding values were significantly higher than expected from genetic drift alone, so that we can conclude that they do result from unintentional selection. This result is congruent with the fact that given the large size of the captive population, the effect of selection could be expected to be stronger than the effects of drift.

The changes in breeding values were strong if considering that they occurred in only four generations. Changes in Haldanes showed that the response of selection was moderate (Table 4) compared with the median of the absolute values for evolutionary rates (5.8 × 10^−3^ standard deviations per generation, Kinnison and Hendry 2001).

It is important to note that the response to selection could not be explained by the dumping strategy because the genetic trends are calculated over generations and not over years. Wild-caught individuals (i.e. as eggs) transferred to the captive breeding will thus be classified as founders (G0). Even under the hypothesis of a massive-biased reinforcement of the wild population, this should result in a smaller difference in life-history traits between the generations (e.g. G0 and G4). However, the speed of these changes could be explained by an acceleration of the response to selection due to genetic correlations. Indeed, all genetic correlations among these traits are positive (Chargé et al. 2013), which could contribute to a faster response to selection. Genetic correlations also most likely explain the surprising change in breeding values of body mass. Body mass is not itself the target of selection but is genetically correlated with traits under strong positive directional selection, leading to a correlated response to selection. However, it is also necessary to scale these changes relatively to the phenotypic values. As such, the changes are important and significant with respect to the scales used in microevolution, but may not imply inevitably a dramatic change in individual phenotypes. For example, the average breeding values for courtship display increased by 1 day, but this has to be related to the mean phenotypic value of 50 days.

### Relaxation of selection

Overall, selection gradients decreased over years for the number of eggs laid. The relaxation of selection is likely due to a better ability of managers to balance families contributions to the next generation when population size is larger. Indeed, captive breeders reached 3600 individuals in 2007 compared with 300 in 1997 (Lesobre 2008). Increased effective population size may have allowed managers to limit the contribution of a few prolific breeders to the next generation. Moreover, improvement of zootechnical practices might have facilitated the expression of poor breeders (i.e. from rare founder genetic lines). Indeed, during the first phase of the captive breeding, managers devoted attention to improve rearing and breeding conditions by limiting stress, improving success of semen collection, enhancing quality of artificial insemination of females, reducing hatching failure of artificially incubated eggs and increasing offspring survival and breeders well-being in captivity. All these improvements of breeding practices led to an increased contribution of poor breeders to the captive population.

There was no evidence of decrease in selection on ejaculate size neither on courtship display (even if selection coefficients were often quite low for courtship display), suggesting that selection was not relaxed on males even after 13 years of breeding. This could be due to a significantly higher reproductive pressure exerted on males compared with females. As one male's semen can be used to inseminate several females, a male producing large ejaculate was likely to sire multiple offspring from several different females, while poor breeders only produce a sufficient amount of sperm to inseminate one female.

### Consequences – genetic diversity

Responses to directional selection raise questions about the maintenance of genetic diversity in the captive broodstock. We could expect a depletion of genetic diversity under these conditions. Contrary to the expectations, initial genetic diversity in the ECWP's captive population has been maintained up to 98% in 2007, mainly thanks to the regular addition of new founders and the large effective population size (Lesobre 2008).

### Consequences – implications for conservation biology

In the Houbara Bustard, we found genetic changes in several life-history traits. However, we would like to discuss the possibility that the consequences of these genetic changes are not necessarily harmful at this stage. Our results show that males with large ejaculate size and high courtship display and more fecund females have been favoured in captivity. In the wild, the species is expected to be under strong sexual selection pressures because of its lek-based mating system (Hingrat et al. 2004). Choosy females are supposed to prefer fertile males that display more (Chargé et al. 2010) to produce fertile and attractive sons and fertile daughters (Chargé et al. 2013). Consequently, we might expect that individuals favoured in captivity could be favoured in the same way in the wild. However, this interpretation deserves further examination as it is also dangerous to jump to the conclusion that genetic changes in captivity increase fitness of individuals both in captivity and in wild harsh conditions, without any fitness evaluation of captive-born individuals released in the wild.

In contrast to the situation in the Houbara captive breeding, a study conducted in *Drosophila melanogaster* showed a dramatic decrease in reproductive fitness (64–86%) after 50 generations, regardless of initial population size, when the populations were moved to ‘wild’ conditions (Woodworth et al. 2002). Similarly, Araki et al. (2007) found that captivity in winter-run steelhead (*Oncorhynchus mykiss*) decreased reproductive success in the wild by 55% between wild-born offspring sired by wild-born parents and a first generation issued from captive-born parent once released in the wild. Another example suggesting a lower fitness in captive-bred individuals in the wild comes from Heath et al. (2003) showing that in a supportive breeding of chinook salmons (*Oncorhynchus tshawytscha*), unintentional selection for female fecundity resulted in smaller eggs size, which was known to reduce early survival. Heavily supplemented wild populations with captive-born salmons also had reduced egg size, which raises serious concerns about the success of captive breeding and supplementation programmes. It is worthwhile to note that all these studies have been conducted on populations that were not under a strict genetic management, contrary to the Houbara's supportive breeding. To the best of our knowledge, we were not aware of similar studies that were investigating the fitness of released captive-born individuals from supportive breeding programmes following worldwide genetic recommendations, such as the equalization of founder genetic lines.

Based on these previous examples, we might expect a fitness reduction in released captive-born Houbaras due to the response to selection in life-history traits. We found in previous experimental studies that more fertile and ‘sexy’ captive males that were able to maintain their courtship activity and sperm quality following an immune challenge sired offspring with a better 1-year survival once released in the wild compared with low-quality males (Chargé et al. 2010, 2011). This result suggests that Houbaras favoured in benign captive conditions were not inevitably maladapted to harsh wild environment where the species lives (i.e. semi-arid areas), contrary to some theoretical predictions (Frankham 2008).

However, the short-term survival of a released population might poorly reflect persistence over the long term (Armstrong 1999). In the Houbara, long-term survival in the wild of captive-born individuals has been shown to be high and similar to wild-born birds and on average higher than short-term survival (<3 months) (Hardouin et al. 2012; L.A. Hardouin, A. Robert, M. Nevoux, O. Gimenez, F. Lacroix, and Y. Hingrat, submitted), suggesting that the higher survival of captive-born Houbara sired by ‘good’ breeders found by Chargé et al. (2011) could reflect a better long-term survival as well.

A recent study investing Houbara breeding parameters in Morocco showed that from the age of two, released and wild females showed similar breeding performances (Bacon 2013). The next step would be to investigate whether offspring sired by the more prolific captive breeders benefit from a higher overall reproductive success in the wild as well.

Overall, despite the growing interest of the use of evolutionary biology for conservation biology (named as ‘Evolutionary Conservation’, Ferrière et al., 2004), very few empirical studies have been addressing the effect of genetic guidelines for supportive breeding on genetic changes, despite quantitative genetics tool available to conduct such investigations in conservation programmes.

The Houbaras' supportive breeding is rather unique in the sense that several thousand individuals are individually managed, while breeding scheme rests on strict genetic guidelines, which might shift the focus on avoiding selection pressures rather than avoiding genetic drift. However, we believe that demographic goals are similar between small and large captive programmes, which may facilitate selection for more prolific breeders, despite that response to selection might change according to the size of captive population. In large supportive programmes (i.e. hundreds of animals), the concerns could be even more important than in the Houbara programme because the excessively large Houbara's captive population may have facilitated relaxation of selection.

To conclude, our results address the question of the success of recognized guidelines for genetic management of captive populations to prevent genetic changes. The answer seems not so straightforward, and definitively, more empirical studies are needed to provide managers with appropriate strategies to preserve, in both captive and supplemented populations, genetic diversity but also genetic quality, as suggested by some authors (Wedekind 2002; Pitcher and Neff 2007).

## Data archiving statement

Breeding values for each individual are available from the Dryad Digital Repository: http://dx.doi.org/10.5061/dryad.b5j25. The raw data underlying the results are not available. We suggest readers to make direct contact with RENECO company.

## References

[b1] Allendorf FW, Luikart G (2007). Conservation and the Genetics of Populations.

[b2] Araki H, Cooper B, Blouin MS (2007). Genetic effects of captive breeding cause a rapid, cumulative fitness decline in the wild. Science.

[b3] Araki H, Cooper B, Blouin MS (2009). Carry-over effect of captive breeding reduces reproductive fitness of wild-born descendants in the wild. Biology Letters.

[b4] Armstrong D (1999). Mortality and behaviour of hihi, an endangered New Zealand honeyeater, in the establishment phase following translocation. Biological Conservation.

[b5] Asa CS, Traylor-Holzer K, Lacy RC (2011). Can conservation-breeding programmes be improved by incorporating mate choice?. International Zoo Yearbook.

[b6] Bacon L (2013).

[b7] Ballou JD, Lacy RC (1995).

[b8] Barnosky AD, Matzke N, Tomiya S, Wogan GOU, Swartz B, Quental TB, Marshal C (2011). Has the Earth's sixth mass extinction already arrived?. Nature.

[b9] Blanchet S, Paez DJ, Bernatchez L, Dodson JJ (2008). An integrated comparison of captive-bred and wild Atlantic salmon (Salmo salar): implications for supportive breeding programs. Biological Conservation.

[b10] Chargé R, Saint Jalme M, Lacroix F, Cadet A, Sorci G (2010). Male health status, signalled by courtship display, reveals ejaculate quality and hatching success in a lekking species. Journal of Animal Ecology.

[b11] Chargé R, Sorci G, Hingrat Y, Lacroix F, Saint Jalme M (2011). Immune-mediated change in the expression of a sexual trait predicts offspring survival in the wild. PLoS One.

[b12] Chargé R, Teplitsky C, Hingrat Y, Saint Jalme M, Lacroix F, Sorci G (2013). Quantitative genetics of sexual display, ejaculate quality and size in a lekking species. Journal of Animal Ecology.

[b13] Duschesne P, Bernatchez L (2002). An analytical investigation of the dynamics of inbreeding in multi-generation supportive breeding. Conservation Genetics.

[b14] Earnhardt JM (1999). Reintroduction programmes: genetic trade-offs for populations. Animal Conservation.

[b15] Ferrière R, Dieckmann U, Couvet D (2004). Evolutionary Conservation Biology.

[b16] Frankham R (2008). Genetic adaptation to captivity in species conservation programs. Molecular Ecology.

[b17] Frankham R, Loebel DA (1992). Modeling problems in conservation genetics using captive Drosophila populations: rapid genetic adaptation to captivity. Zoo Biology.

[b18] Frankham R, Manning H, Margan SH, Briscoe DA (2000). Does equalization of family sizes reduce genetic adaptation to captivity?. Animal Conservation.

[b19] Fraser DJ (2008). How well can captive breeding programs conserve biodiversity? A review of salmonids. Evolutionary Applications.

[b20] Gelman A (2006). Prior distributions for variance parameters in hierarchical models (comment on article by Browne and Draper). Bayesian Analysis.

[b21] Goriup PD (1997). The world status of the Houbara Bustard *Chlamydotis undulata*. Bird Conservation International.

[b22] Grahn M, Langefors A, von Schantz T, Caro TM (1998). The importance of mate choice in improving viability in captive populations. Behavioral Ecology and Conservation Biology.

[b23] Hadfield JD, Wilson AJ, Garant D, Sheldon BC, Kruuk LEB (2010). The misuse of BLUP in ecology and evolution. The American Naturalist.

[b24] Hardouin LA, Nevoux M, Robert A, Gimenez O, Lacroix F, Hingrat Y (2012). Determinants and costs of natal dispersal in a lekking species. Oikos.

[b500] Heath DD (2003). Rapid evolution of egg size in captive Salmon. Science.

[b25] Hingrat Y, Saint Jalme M, Ysnel F, Lacroix F, Seabury J, Rautureau P (2004). Relationships between home-range-size, sex and season with preference to the mating system of the Houbara Bustard *Chlamydotis undulata* undulata. Ibis.

[b26] Hingrat Y, Saint Jalme M, Chalah T, Orhant N, Lacroix F (2008). Environmental and social constraints on breeding site selection. Does the exploded-lek and hotspot model apply to the Houbara Bustard *Chlamydotis undulata* undulata?. Journal of Avian Biology.

[b27] Kinnison MT, Hendry AP, Hendry AP, Kinnison MT (2001). The pace of modern life II: from rates of contemporary microevolution to pattern and process. Microevolution Rate, Pattern, Process.

[b28] Kruuk LEB (2004). Estimating genetic parameters in natural populations using the “animal model”. Philosophical Transactions of the Royal Society of London. Series B: Biological Sciences.

[b29] Lacroix F (2003). The Emirates Center for Wildlife Propagation: developing a comprehensive strategy to secure a self sustaining population of Houbara Bustards in eastern Morocco. Houbara News.

[b30] Lacy RC (2000). Should we select genetic alleles in our conservation breeding programs?. Zoo Biology.

[b31] Lake PE, Ravie O (1984). An exploration of cryoprotective compounds for fowl spermatozoa. British Poultry Science.

[b32] Lande R, Arnold SJ (1983). The measurement of selection on correlated characters. Evolution.

[b33] Lesobre L (2008).

[b34] Lynch M (1998). Genetics and Analysis of Quantitative Traits.

[b35] Lynch M, O'Hely M (2001). Captive breeding and the genetic fitness of natural populations. Conservation Genetics.

[b36] Pelletier F, Réale D, Watters J, Boakes EH, Garant D (2009). Value of captive populations for quantitative genetics research. Trends in Ecology & Evolution.

[b37] Pimm SL, Russell GJ, Gittleman JL, Brooks TM (1995). The future of biodiversity. Science.

[b38] Pitcher TE, Neff BD (2007). Genetic quality and offspring performance in Chinook salmon: implications for supportive breeding. Conservation Genetics.

[b39] Preston BT, Jalme MS, Hingrat Y, Lacroix F, Sorci G (2011). Sexually extravagant males age more rapidly. Ecology Letters.

[b40] Ralls K, Ballou JD, Rideout BA, Frankham R (2000). Genetic management of chondrodystrophy in California condors. Animal Conservation.

[b41] Reisenbichler RR, Rubin SP (1999). Genetic changes from artificial propagation of Pacific salmon affect the productivity and viability of supplemented populations. ICES Journal of Marine Science: Journal du Conseil.

[b42] Saint Jalme M, Gaucher P, Paillat P (1994). Artificial insemination in Houbara Bustards (*Chlamydotis undulata*): influence of the number of spermatozoa and insemination frequency on fertility and ability to hatch. Journal of Reproduction and Fertility.

[b43] Saint Jalme M, Combreau O, Seddon PJ, Paillat P, Gaucher P, Van Heezik Y (1996). Restoration of *Chlamydotis undulata* macqueenii (Houbara Bustard) populations in Saudi Arabia: a progress report. Restoration Ecology.

[b44] Saint Jalme M, Lecoq R, Seigneurin F, Blesbois E, Plouzeau E (2003). Cryopreservation from semen of endangered pheasants: the first step towards a cryobank for endangered avian species. Theriogenology.

[b45] Saura M, Pérez-Figueroa A, Fernández J, Toro MA, Caballero A (2008). Preserving population allele frequencies in ex situ conservation programs. Conservation Biology.

[b46] Seddon PJ, Soorae PS, Launay F (2005). Taxonomic bias in reintroduction projects. Animal Conservation.

[b47] Wang J, Ryman N (2001). Genetic effects of multiple generations of supportive breeding. Conservation Biology.

[b48] Wedekind C (2002). Sexual selection and life-history decisions: implications for supportive breeding and the management of captive populations. Conservation Biology.

[b49] Williams SE, Hoffman EA (2009). Minimizing genetic adaptation in captive breeding programs: a review. Biological Conservation.

[b51] Woodworth LM, Montgomery ME, Briscoe DA, Frankham R (2002). Rapid genetic deterioration in captive populations: causes and conservation implications. Conservation Genetics.

