# Supplementary material for: Does recognized genetic management in supportive breeding prevent genetic changes in life-history traits?
Source: Evol Appl. 2014 Mar 27;7(5):521–32. doi: 10.1111/eva.12150 (PMC4055174; doi:10.1111/eva.12150)

SUPPLEMENTARY MATERIAL

Table S1: Basic statistics describing the pedigree. The pedigree has been pruned in order to retain only individuals informative for the traits under study (function *pedantics* in R, Morrissey & Wilson, 2010).

	Number of days with display	Number of eggs	Ejaculate size	Female body mass	Male body mass
Number of records	4591	6247	3472	6080	4495
Number of founders	226	240	185	237	221
Number of maternities	4352	6002	3267	5811	4261
Number of paternities	4323	5973	3248	5838	4236
Pedigree depth	8	9	8	9	8

Table S2: Annual selection differentials and gradients (± SE) for all years investigated in the study. Significance levels come from a model performed on non-standardized values of number of chicks with a Poisson distribution of errors. * p<0.05, ** p<0.01, *** p<0.001.

	Selection differentials					Selection gradients				
Year	Display rate	Ejaculate size	Male body mass	Number of eggs	Female body mass	Display rate[a]	Ejaculate size[a]	Male body mass[a]	Number of eggs[b]	Female body mass[b]
1999	*0.46 ± 0.13 ***			*1.41 ± 0.15 ****	*0.59 ± 0.19 ****				*1.36 ± 0.18 ****	*0.12 ± 0.19*
2000	*0.71 ± 0.38 ****	*1.26 ± 0.22 ****	*-1.28 ± NA ****	*0.97 ± 0.07 ****	*0.58 ± 0.10 ****	*-4.02 ± NA*	*1.52 ± NA ****	*NA*	*0.90 ± 0.08 ****	*0.13 ± 0.08*
2001	*0.14 ± 0.16*	*0.32 ± 0.15 ***	*0.02 ± 0.13*	*1.03 ± 0.06 ****	*0.56 ± 0.10 ****	*0.10 ± 0.17*	*0.18 ± 0.16*	*-0.01 ± 0.13*	*0.99 ± 0.07 ****	*0.10 ± 0.07*
2002	*0.38 ± 0.19 ****	*0.60 ± 0.19 ****	*0.28 ± 0.17 ****	*0.79 ± 0.06 ****	*0.44 ± 0.08 ****	*0.38 ± 0.18 ****	*0.57 ± 0.19 ****	*0.18 ± 0.17 ****	*0.74 ± 0.07 ****	*0.19 ± 0.07 ****
2003	*0.38 ± 0.12 ****	*0.59 ± 0.13 ****	*-0.02 ± 0.13*	*0.82 ± 0.06 ****	*0.21 ± 0.08 ****	*0.33 ± 0.11 ****	*0.56 ± 0.12 ****	*-0.08 ± 0.11*	*0.80 ± 0.06 ****	*0.12 ± 0.07 **
2004	*0.22 ± 0.09 ****	*0.27 ± 0.10 ****	*-0.05 ± 0.09*	*0.70 ± 0.04 ****	*0.21 ± 0.05 ****	*0.19 ± 0.09 ****	*0.28 ± 0.10 ****	*-0.11 ± 0.09 ***	*0.67 ± 0.04 ****	*0.12 ± 0.04*
2005	*0.19 ± 0.08 ****	*0.19 ± 0.08 ****	*-0.19 ± 0.09 ****	*0.67 ± 0.06 ****	*0.18 ± 0.06 ****	*0.15 ± 0.08 ***	*0.18 ± 0.08 ****	*-0.17 ± 0.09 ***	*0.66 ± 0.06 ****	*0.09 ± 0.06*
2006	*0.11 ± 0.12 ***	*0.41 ± 0.11 ****	*0.01 ± 0.12*	*0.48 ± 0.08 ****	*0.12 ± 0.08 ***	*0.10 ± 0.12 **	*0.42 ± 0.11 ****	*-0.06 ± 0.12*	*0.49 ± 0.08 ****	*0.00 ± 0.08*
2007	*-0.03 ± 0.13*	*0.75 ± 0.10 ****	*0.17 ± 0.13 ****	*0.45 ± 0.06 ****	*0.11 ± 0.06 ****	*-0.07 ± 0.12*	*0.75 ± 0.10 ****	*0.10 ± 0.12 ****	*0.45 ± 0.06 ****	*-0.01 ± 0.06*
2008	*0.20 ± 0.10 ****	*0.61 ± 0.09 ****	*0.11 ± 0.08 ****	*0.88 ± 0.05 ****	*0.23 ± 0.05 ****	*0.24 ± 0.09 ****	*0.62 ± 0.09 ****	*0.03 ± 0.08*	*0.88 ± 0.05 ****	*-3.73.10-3 ± 0.08*
2009	*0.04 ± 0.10*	*0.66 ± 0.09 ****	*0.07 ± 0.09 ***	*0.45 ± 0.05 ****	*0.18 ± 0.05 ****	*0.03 ± 0.10*	*0.71 ± 0.09 ****	*-0.01 ± 0.08*	*0.44 ± 0.05 ****	*0.06 ± 0.05 **
2010	*0.19 ± 0.17 ****	*0.40 ± 0.16 ****	*0.14 ± 0.16 ***	*0.47 ± 0.09 ****	*-0.08 ± 0.09*	*0.19 ± 0.17 ****	*0.40 ± 0.16 ****	*0.07 ± 0.16*	*0.54 ± 0.09 ****	*-0.25 ± 0.09 ****
2011	*0.14 ± 0.12 ****	*0.30 ± 0.12 ****	*0.09 ± 0.12 **	*0.42 ± 0.06 ****	*-0.10 ± 0.06 ***	*0.13 ± 0.12 ***	*0.30 ± 0.12 ****	*0.05 ± 0.12*	*0.49 ± 0.07 ****	*-0.24 ± 0.07 ****

[a] selection gradients from models in which display rate, ejaculate size and male body mass have been set as covariates
[b] selection gradients from models in which number of eggs and female body mass have been set as covariates

Table S3: Time trends in annual selection gradients. Table presents the estimates with 95% confidence interval.

	Trend in selection gradients [95%CI]	Probability to be equal to 0
Courtship display	0.11 [-4.50; 3.61]	0.52
Ejaculate size	0.02 [-0.03; 0.06]	0.4
Number of eggs	-0.05 [-0.06; -0.02]	**<0.005**
Female body mass	-0.10 [-1.00; 1.03]	0.29
Male body mass	2.24 [-8.77; 4.38]	0.58

Table S4: Time trends in breeding values estimated either with a parameter expanded prior (a) or a slightly informative prior (b) (V=diag(n)*Vp/r, nu = n, where Vp is the phenotypic variance, n the number of traits and r the number of random factors). P_T and P_D: respectively probability of the posterior distribution for the estimate being equal to zero, and similar from expectation under the hypothesis of drift only.

(a)

	Trend in breeding values		.
	estimate [95%CI]	P_T	P_D
Display rate	0.27 [0.21; 0.42]	0	0
Ejaculate size	0.17 [0.07; 0.31]	0	0.024
Number of eggs	0.21 [0.18; 0.30]	0	0
Female body mass	19.04 [14.67; 21.86]	0	0.001
Male body mass	37.35 [29.34; 44.89]	0	0.001

(b)

	Trend in breeding values		.
	estimate [95%CI]	P_T	P_D
Display rate	0.29 [0.21; 0.41]	0	0
Ejaculate size	0.25 [0.16; 0.40]	0	0.001
Number of eggs	0.22 [0.16; 0.28]	0	0
Female body mass	18.98 [14.67; 22.31]	0	0
Male body mass	37.92 [30.35; 45.37]	0	0

Table S5: fixed age effects (a) and random maternal effects (b) on the assessment of the genetic additive variance. Confidence intervals are indicated between square brackets. Variances were estimated with a parameter expanded prior. Models aiming at assessing dam effects included age and age² as fixed effects (see Table S5a for the model excluding dam effects), while all models included year of sampling, animal and permanent environment as random effects.

(a)

	Animal model including age and age² Va [95%CI]	Animal model excluding age and age² Va [95%CI]
Display rate	0.22 [0.17; 0.29]	0.29 [0.23; 0.35]
Ejaculate size	0.28 [0.20; 0.38]	0.25 [0.18; 0.32]
Number of eggs	0.19 [0.15; 0.24]	0.13 [0.11; 0.18]
Female body mass	10726.32 [9574.75; 12257.97]	11233.91 [9933.92; 12807.67]
Male body mass	21122.71 [18118.79; 24723.46]	18728.01 [15280.31; 21988.97]

(b)

	Animal model including random dam effects	
	Va [95%CI]	Vdam [95%CI]
Display rate	0.24 [0.16; 0.31]	$1.85*10^{-4}$ [$8.03*10^{-8}$; 0.03]
Ejaculate size	0.28 [0.19; 0.38]	$1.61*10^{-4}$ [$6.59*10^{-7}$; 0.04]
Number of eggs	0.16 [0.12; 0.21]	$2.94*10^{-5}$ [$2.98*10^{-10}$; 0.01]
Female body mass	11493.61 [10028.98; 13134.81]	273.23 [$3.72*10^{-3}$; 659.33]
Male body mass	21140.87 [16516.63; 24020.44]	2016.14 [578.32; 2911.36]

Figure S1: fixed age effects (a) and random maternal effects (b) on the assessment of the genetic additive variance. Error bars refer to confidence intervals.

(a)

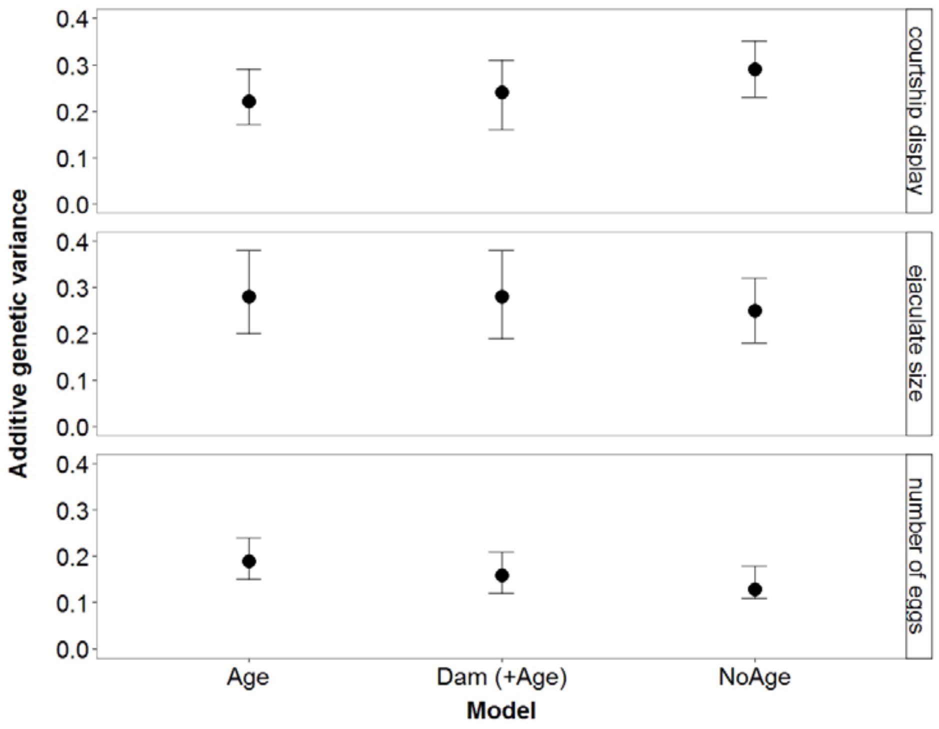

(b)

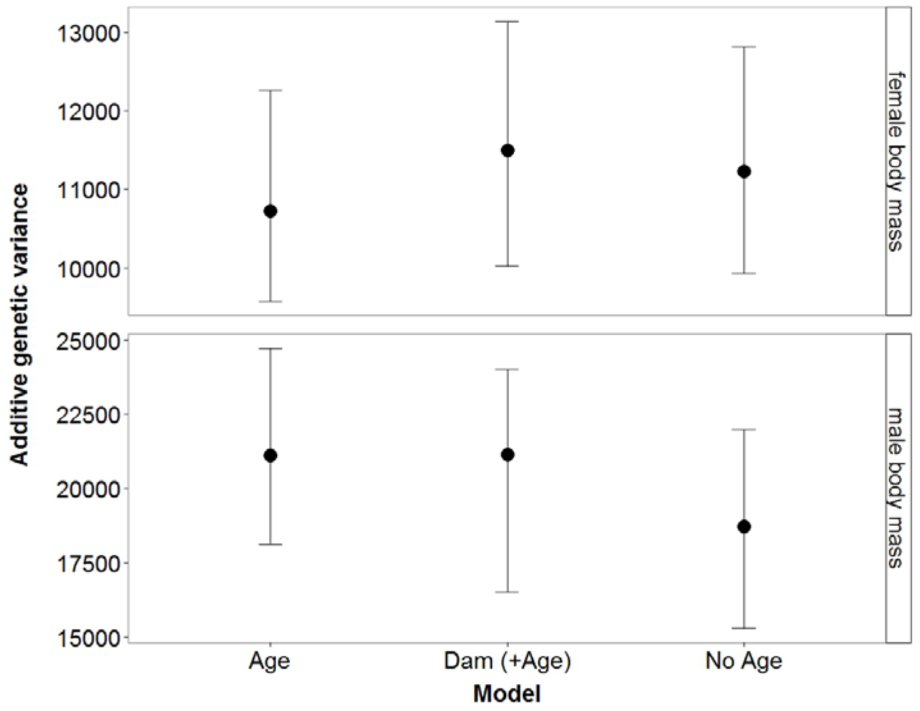

Supplement: Supplementary file 1 [file eva0007-0521-SD1.pdf]
